# Dual endothelin receptor inhibition enhances T-DM1 efficacy in brain metastases from HER2-positive breast cancer

**DOI:** 10.1038/s41523-018-0100-8

**Published:** 2019-01-15

**Authors:** Vasileios Askoxylakis, Gino B. Ferraro, Mark Badeaux, David P. Kodack, Isabelle Kirst, Ram C. Shankaraiah, Christina S. F. Wong, Dan G. Duda, Dai Fukumura, Rakesh K. Jain

**Affiliations:** Edwin L. Steele Laboratories, Department of Radiation Oncology, Massachusetts General Hospital and Harvard Medical School, Boston, MA USA

## Abstract

The effective treatment of cerebral metastases from HER2-positive breast cancer remains an unmet need. Recent studies indicate that activated astrocytes and brain endothelial cells exert chemoprotective effects on cancer cells through direct physical interaction. Here we report that the endothelin axis mediates protection of *HER2*-amplified brain metastatic breast cancers to the anti-HER2 antibody–drug conjugate ado-trastuzumab emtansine (T-DM1). Macitentan, a dual inhibitor of endothelin receptors A and B, improves the efficacy of T-DM1 against breast cancers grown in the brain. We show that direct contact of brain stroma with cancer cells is required for protection to T-DM1. Our data suggest that targeting the endothelin axis may be beneficial when anti-signaling agent and cytotoxic agent are combined. These findings may contribute to the development of therapeutic approaches with enhanced efficacy in the brain microenvironment.

## Introduction

The brain microenvironment has a key role in metastatic tumor progression. Microglia, activated astrocytes, and brain endothelial cells may support survival and growth of brain metastases (BM).^1^ Studies indicate that astrocytes and brain endothelial cells have chemoprotective effects on cancer cells through an endothelin pathway-mediated mechanism.^2^ Specifically, physical interaction between metastatic cancer cells and brain stromal cells increased expression of endothelins (ET) in astrocytes, activating the endothelin pathway in cancer cells and promoting their survival.^2^ Treatment with macitentan, a dual inhibitor of ET_A_ and ET_B_ receptors abolished the astrocyte- and endothelial cell-mediated chemoprotection, resulting in apoptosis increase after paclitaxel chemotherapy in brain metastasis models from breast and lung cancer.^3^ We recently demonstrated that the antibody–drug conjugate ado-trastuzumab emtansine (T-DM1) has preclinical activity against BM from HER2-positive breast cancer.^4^ T-DM1 increased mitotic catastrophe through the DM1 component, facilitated by selective trastuzumab-mediated targeting. Whether endothelin pathway activation plays any role in this setting is unknown. We hypothesized that dual ET_A_ and ET_B_ inhibition can enhance the activity of T-DM1 against BM from HER2-positive breast cancer.

## Results

**Fig. 1:**
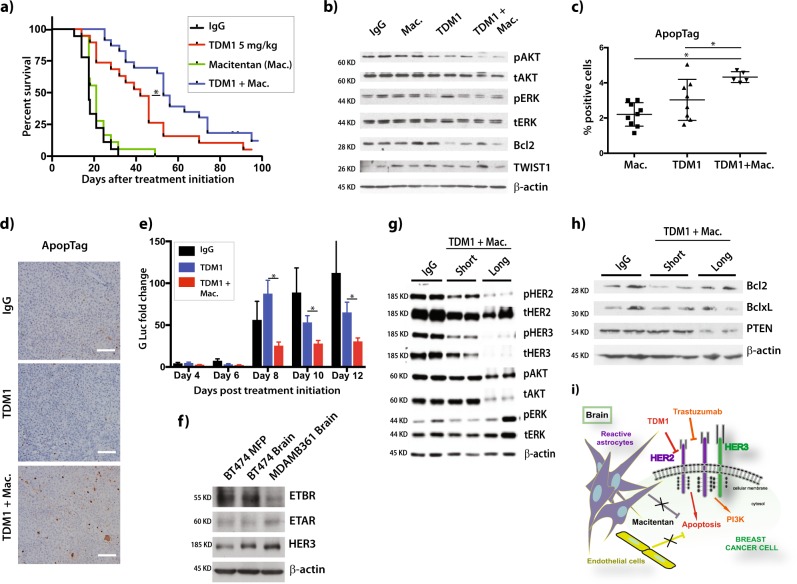
Effects of macitentan on the efficacy of T-DM1 against HER2-positive breast cancer tumors in the brain microenvironment. **a** Mouse survival in nude mice with BT474-Gluc brain tumors treated with i) control vehicle, T-DM1 (5 mg/kg, i.v. weekly), macitentan (50 mg/kg p.o. daily) or their combination (N = 18-21). **b** Western blots were performed in BT474-Gluc tumors, collected 48 h after treatment (blot samples were derived from the same experiment and were processed in parallel). **c** Cell apoptosis was determined in BT474-Gluc brain metastases after quantification of ApopTag, 5 days after treatment with control vehicle, T-DM1 (1 × 5 mg/kg), or T-DM1 (1 × 5 mg/kg) + macitentan (5 × 50 mg/kg) (N = 5-9). Error bars are standard deviation. **d** Representative images of BT474-Gluc tumors in the brain after staining for ApopTag. Scale bar = 0.1 mm. **e** Relative activity of Gaussia luciferase in the media of organotypic brain slice cultures of BT474-Gluc cells following treatment with T-DM1 alone or in combination with macitentan. Gluc levels from treated cells were normalized to Day 0 for each treatment. Error bars are standard deviation (*N* = 5–7). **f** Western blots were performed in BT474-Gluc tumors growing in the brain or the mammary fat pad (MFP), and in MDA-MD-361-Gluc brain tumors. HER3 was used as control, since it has been previously established that the brain microenvironment increases HER3 expression^6^ **g**, **h**. Western blots were performed in BT474-Gluc brain tumors, collected 48 h after treatment with T-DM1 + macitentan (T-DM1 + mac. short) and at the endpoint of the study (T-DM1 + mac. long). Tumors from mice treated with unspecific IgG were collected and used as control. **i** Schematic of brain-stroma mediated protection to the HER2-targeted ADC T-DM1. Direct contact of reactive astrocytes and brain endothelial cells with *HER2*-amplified breast cancer cells can reduce the activity of ado-trastuzumab emtansine (T-DM1) through an endothelin-pathway-mediated mechanism. Dual endothelin receptor inhibition with macitentan increases T-DM1 induced apoptosis and enhances the activity of the ADC in the brain microenvironment

We treated mice bearing human *HER2*-amplified BT474 brain tumors, engineered to express secretable Gaussia luciferase (BT474-Gluc),^4^ with T-DM1 alone or in combination with macitentan. Adding macitentan to T-DM1 delayed brain tumor growth. Median mouse survival improved from 42 days in the T-DM1 group to 53 days for T-DM1 plus macitentan (HR = 0.79, *p* = 0.042) (Fig. 1a).^5^ Macitentan monotherapy did not affect tumor growth or survival. We next investigated the mechanism of synergy between macitentan and T-DM1. Western blot analysis of protein extracted from BT474-Gluc BM three days after treatment initiation revealed decreased AKT phosphorylation, as well as decreased expression of the anti-apoptotic protein Bcl2 (Fig. 1b).^5^ Significant apoptosis increase was found in the T-DM1 plus macitentan group (one-way ANOVA, *p* < 0.001) (Fig. 1c, d).^5^

Previous reports suggested that physical interaction between cancer cells and brain-stromal cells is required for endothelin-mediated chemoprotection.^2^ We used organotypic brain-slice cultures to evaluate the role of cell-cell contact (Supplemental Fig. 1A).^5^ BT474-Gluc cells were either injected directly onto organotypic brain slices or grown in brain-slice conditioned media. Adding macitentan to T-DM1 delayed BT474-Gluc growth significantly, when growing in direct contact with the brain slice (Fig. 1e).^5^ However, when cancer cells were exposed to brain-slice conditioned media without physical contact, macitentan did not enhance T-DM1 activity (Supplemental Fig. 1B-C).^5^ These data confirm that direct contact with cancer cells is required for stromal cell-mediated protection.

To determine molecular changes in vivo, we investigated ET_A_ and ET_B_ expression in BT474-Gluc tumors implanted in the brain or mammary fat pad (MFP), as well as in breast cancer MDA-MB-361-Gluc brain tumors. As expected, HER3 was overexpressed in brain samples (Fig. 1f).^5,6^ No clear difference in endothelin receptor expression between the isogenic BT474-Gluc tumors in brain and MFP was seen (Fig. 1f).^5^ However, we observed differential expression of ET_A_ and ET_B_ between BT474-Gluc and MDA-MB-361-Gluc brain tumors. Moreover, we investigated HER2-downstream signaling three days after treatment initiation, when cells were sensitive to T-DM1 plus macitentan, and at the endpoint of the study, after cells became treatment-resistant. Western blot revealed a decrease in expression and phosphorylation of HER2 and HER3, compared to control on day 3 (Fig. 1g).^5^ Of note, a striking decrease in HER2 and HER3 expression and activation was noticed at the study endpoint (Fig. 1g).^5^ Short-term treatment with T-DM1 alone did not clearly affect HER2 phosphorylation, compared to control. However, HER2 and HER3 were also strongly decreased at the study endpoint (Supplemental Fig. 1D-E).^5^ Despite a decrease of total AKT for the combination of T-DM1 with macitentan at the endpoint, AKT phosphorylation was still active, whereas ERK signaling remained activated (Fig. 1g).^5^ Moreover, Bcl2 expression was restored in tumors resistant to T-DM1 plus macitentan (Fig. 1h).^5^ These effects were associated with a strong PTEN reduction (Fig. 1h).^5^ PTEN regulates pro-survival signaling,^7^ and mediates Bcl2 expression at the translational level.^8^ The brain microenvironment can promote PTEN loss in cancer cells.^9^ Thus, PTEN loss may promote resistance of BT474-Gluc brain tumors to treatment with T-DM1 plus macitentan.

We next investigated the effects of dual endothelin-receptor inhibition on the efficacy of HER2-signaling inhibition. We combined macitentan with the HER2/EGFR small-molecule inhibitor neratinib. The rationale for choosing neratinib was two-fold: i) it inhibits irreversibly erbB1, HER2 and erbB4, and ii) data suggest drug penetration across the BBB.^10^ Our studies showed minimal tumor growth delay for neratinib alone (Supplemental Fig. 1F),^5^ which is not-surprising, since HER2-targeted therapies have shown limited efficacy against BM.^6,11^ Adding macitentan to neratinib did not improve survival, compared to control-treated mice (Supplemental Fig. 1G).^5^ These data support the hypothesis that the role of the endothelin axis may be more specific to chemoprotection in BM.

T-DM1 was effective against brain metastases from *HER2*-amplified breast cancer independent of the *PIK3CA* mutation status.^4^ We investigated the effects of adding macitentan to T-DM1 on its efficacy against MDA-MB-361-Gluc BM, which are *HER2*-amplified but also harbor an activating *PIK3CA* mutation (E545K). Our studies confirmed the efficacy of T-DM1, however, adding macitentan did not improve its activity (Supplemental Fig. 1H).^5^ Similar to the BT474 model, comparison of HER2 signaling revealed a decrease in HER2 and HER3 expression and phosphorylation after the MDA-MD-361-Gluc tumors became resistant to treatment. Of note, AKT and ERK phosphorylation were enhanced in MDA-MB-361 tumors (Supplemental Fig. 1I),^5^ an effect that was stronger when macitentan was added to T-DM1 (Supplemental Fig. 1I).^5^ The underlying mechanisms of the increased signaling in response to dual endothelin-receptor inhibition in MDA-MB-361 brain tumors remain unclear. Endothelin receptor expression analysis of MDA-MD-361-Gluc brain tumors showed decreased expression of ET_B_, compared to BT474-Gluc brain tumors. This could provide an additional explanation for the lack of activity of macitentan, allowing the hypothesis that endothelin-mediated resistance in the brain microenvironment may not only be mediated by homo– and hetero-receptor expression and compensatory oncogenic downstream signaling, but also by receptor activation through increased ligand expression by brain stromal cells. Indeed previous studies show that peritumoral astrocytes overexpress endothelins in more than 80% of human brain metastases.^12^ Collectively, these data indicate that the effects of dual endothelin-receptor inhibition may vary between tumors with different characteristics, thus underscoring the need for personalized treatment.

## Discussion

In conclusion, our studies show a potential role of the endothelin axis in protection of *HER2*-amplified brain metastatic cells to an anti-HER2 antibody–drug-conjugate (Fig. 1i). We show in clinically relevant brain-metastasis models that dual endothelin-receptor inhibition improved T-DM1 activity, and that this effect required direct contact of brain-stroma with cancer cells. Dual endothelin-receptor blockade did not overcome resistance to a HER2-signaling inhibitor in the brain microenvironment in the absence of cytotoxic treatments. Conversely, it did not improve sensitivity to cytotoxic therapy in *HER2*-amplified brain metastases with activating *PIK3CA* mutation, characterized by absence of HER2-downstream signaling inhibition. These findings suggest that the beneficial effects of endothelin inhibitors on T-DM1 involve both the inhibition of intrinsic signaling pathways and the cytotoxic component. Considering that effective treatment of brain metastases from HER2-positive breast cancer remains an unmet need, our findings may contribute to the development of therapeutic approaches with enhanced efficacy in the brain microenvironment. Further evaluation of endothelin signaling and the impact of endothelin-receptor inhibition in brain metastases from HER2-positive breast cancer is warranted.

## Methods

### Cell lines

Human *HER2*-amplified breast cancer BT474 cells (ATCC) were cultured in RPMI 1640 supplemented with 10% FBS (Atlanta Biologicals, Flowery Branch, GA, USA). Human *HER2*-amplified MDA-MB-361 cells (ATCC) were cultured in DMEM/F12 supplemented with 10% FBS. BT474 and MDA-MB-361 cells were transduced with an expression cassette encoding Gaussia luciferase (Gluc) and green fluorescent protein (GFP), as previously described.^6^

### Animal models

A total of 100,000 BT474-Gluc or MDA-MB-361-Gluc cells diluted in 1 μL PBS were implanted in the frontal brain lobe of 8-week-old female nude mice as previously described.^4,6^ All animal procedures were performed according to guidelines of the Public Health Service Policy on Human Care of Laboratory Animals and in accordance with a protocol approved by the Institutional Animal Care and Use Committee of Massachusetts General Hospital.

### Reagents and treatments

T-DM1 and nonspecific human IgG (Jackson ImmunoResearch Laboratories, Inc., West Grove, PA) were administered weekly at a concentration of 5 mg/kg body weight i.v. Macitentan was administered at a dose of 50 mg/kg daily per os.

### Organotypic brain slice cultures

A 300-µm thick brain slices were obtained from postnatal day 17-20 mice using a Compresstome™ Vf-300 microtome (Precisionary Instruments, Greenville, NC). Slices were cultured into inserts with 50% MEM, 25% EBSS, 25% HS, Gentamycin and Glucose (Invitrogen Life Technologies, Grand Island, NY). 7,500 BT474-Gluc cells were injected into the cortical layers 4–6 days after slice preparation. Media was collected every 2 days and Gluc activity was measured with a Promega Glomax 96 microplate luminometer (Fisher Scientific, Waltham, MA).

### Immunostaining

Apoptag (ApopTag® Peroxidase In Situ Apoptosis Detection Kit, #S7100, Millipore) was used as apoptosis marker. Paraffin embedded tissue (*N* = 5-6), was sectioned and stained for DNA fragmentation by direct TUNEL method (ApopTag® Peroxidase In Situ Apoptosis Detection Kit, #S7100, Millipore), as previously described.^4^

### Western blotting

Protein was collected from tumors 48 h after treatment or at the endpoint of the study. All primary antibodies were obtained from Cell Signaling Technologies, Danvers, MA. Primary antibodies (diluted 1:1000) included monoclonal rabbit antibodies for HER2 (Cat#2165), pHER2 (Cat#2243), Akt (Cat#4691), pAkt (Cat#4060), Erk1/2 (Cat#9102), pErk1/2 (Cat#4370). HER3 (Cat#12708), pHER3 (Cat#2842), Bcl2 (Cat#2872), BclXL (Cat#2754), TWIST (Cat#46702), PTEN (Cat#9188). ETAR and ATBR antibodies were obtained from Santa Cruz. All blots derive from the same experiment and processed in parallel for each respective panels. Beta-actin was used as internal control.

### Statistical analysis

Statistical analysis was performed using Prism 6 (GraphPad Software Inc., La Jolla, CA). All statistical tests were two-sided. A difference was considered statistically significant when *P* < 0.05. Comparisons of continuous outcomes were performed using one-way ANOVA test with Bonferroni-Holms correction for categorical and *t* test for dichotomous variables. Kaplan–Meier analysis and log-rank test were used for survival analysis. Measurements were taken from distinct samples, except from in vivo tumor growth measurements, where the same sample was measured repeatedly.

## Supplementary information

Supplemental file

## Data Availability

The raw image and graph data generated and analysed during this study are described in the following data record: 10.6084/m9.figshare.7462034. All the data files are available on request from the R.K.J. at the following email: PAtoDrJain@steele.mgh.harvard.edu.

## **Supplementary information**

**Supplementary information** accompanies the paper on the *npj Breast Cancer* website (10.1038/s41523-018-0100-8).
