# Supplementary material for: Dual endothelin receptor inhibition enhances T-DM1 efficacy in brain metastases from HER2-positive breast cancer
Source: NPJ Breast Cancer. 2019 Jan 15;5:4. doi: 10.1038/s41523-018-0100-8 (PMC6333771; doi:10.1038/s41523-018-0100-8)

Supplemental Figure 1.

(A) Representative confocal image of organotypic brain slice culture co-cultured with BT474-GFP cells for 10 days. Immunofluoresence: Glial fibrillary acidic protein (GFAP) antibody to label astrocytes; GFP and DAPI labelling of BT474 cells Scale bar = 0.05mm. **(B)** Relative activity of Gaussia luciferase in the media of BT474-Gluc cells following treatment with T-DM1 or T-DM1 plus macitentan in horse serum media (HS) from organotypic slice cultures for 4 days and 8 days. Gluc levels from treated cells were normalized to Day 0 for each treatment. Error bars are standard deviations (N=2). **(C)** Relative activity of Gaussia luciferase in the media of BT474-Gluc cells following treatment with T-DM1 or T-DM1 plus macitentan in conditioned media (CM) from organotypic slice cultures for 4 days and 8 days. Gluc levels from treated cells were normalized to Day 0 for each treatment. Error bars are standard deviations (N=2). **(D)** Western blots were performed in BT474-Gluc tumors, collected 48 hours after treatment with T-DM1 alone (T-DM1 short) and at the endpoint of the study (T-DM1 long). Tumors from mice treated with unspecific IgG were collected and used as control (blot samples were derived from the same experiment and were processed in parallel). **(E)** Average (n=2) fold intensity change to respective controls for western bands from Figure 1G (top) and Supplemental Figure 1D (bottom). Tumor growth **(F)** and mouse survival (N=3-5) **(G)** in nude mice with BT474-Gluc brain tumors treated with control vehicle, neratinib (20 mg/kg, i.v. daily), macitentan (50 mg/kg p.o. daily) or their combination. Blood Gaussia luciferase activity (Relative Light Units per sec, RLU/s) was used as surrogate for tumor growth. **(H)** Survival of mice bearing HER2-amplified PIK3CA mutant (E545K) MDA-MB-361-Gluc tumors (N=6). **(I)** HER2-downstream signaling in MDA-MB-361-Gluc brain tumors, collected at the endpoint of the study following treatment with T-DM1 at 5 mg/kg i.v. weekly (T-DM1 long) or T-DM1 at 5 mg/kg i.v. weekly in combination with macitentan at 50 mg/kg p.o. daily (T-DM1 + mac. long). Brain tumors from mice treated with unspecific IgG were collected and used as control (blot samples were derived from the same experiment and were processed in parallel).

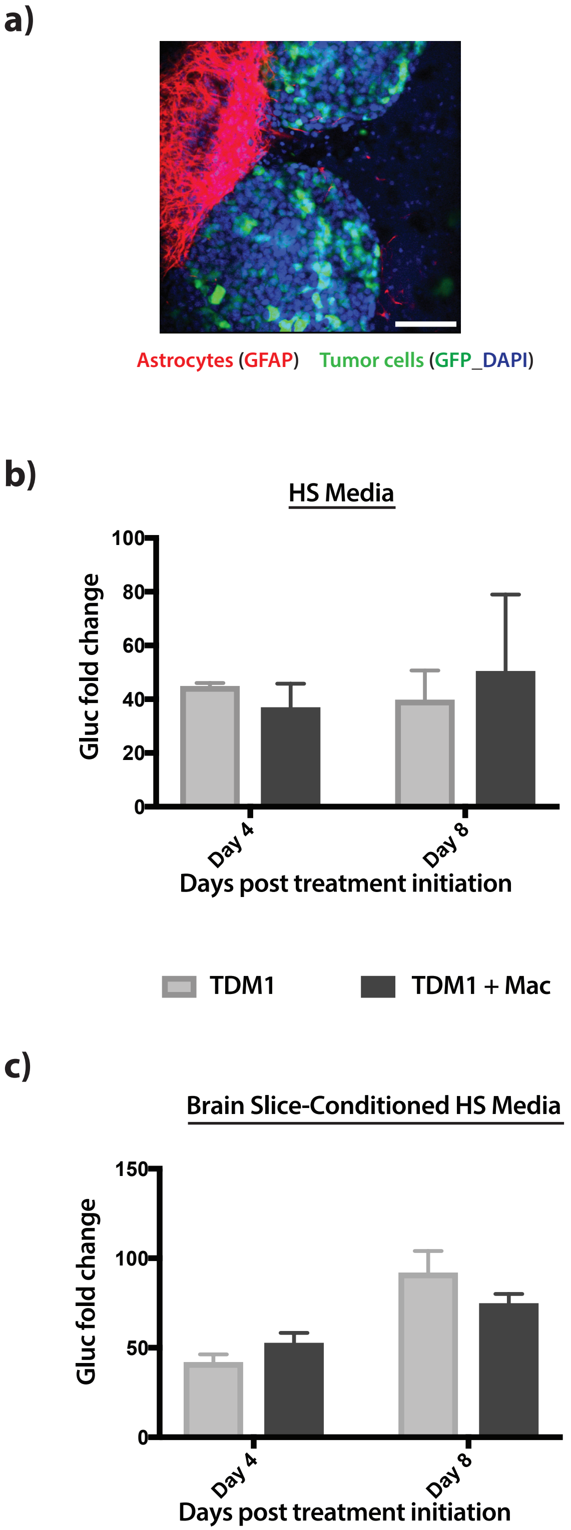

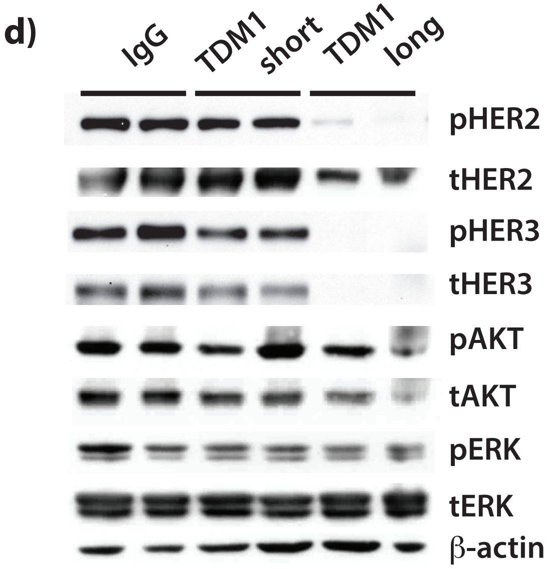

e)

Fold change (△control) - *Fig 1G*	
pHER2	0.51
tHER2	0.68
pHER3	0.33
tHER3	0.64
**Fold change (△control) - Suppl. *Fig 1D***	
pHER2	0.79
tHER2	1.3
pHER3	0.7
tHER3	0.63

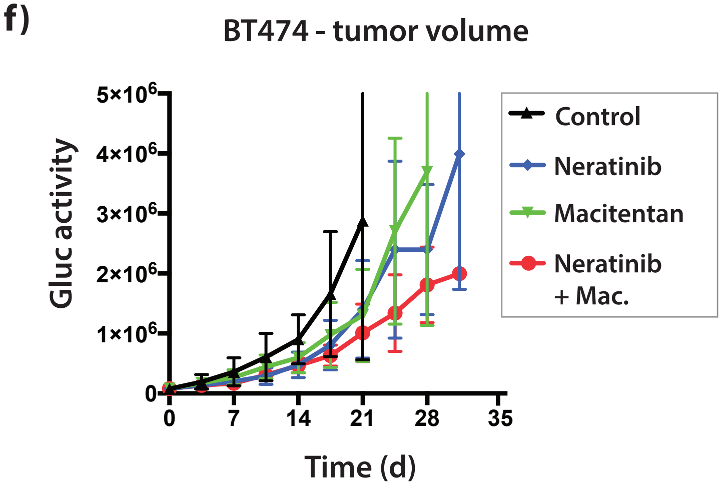

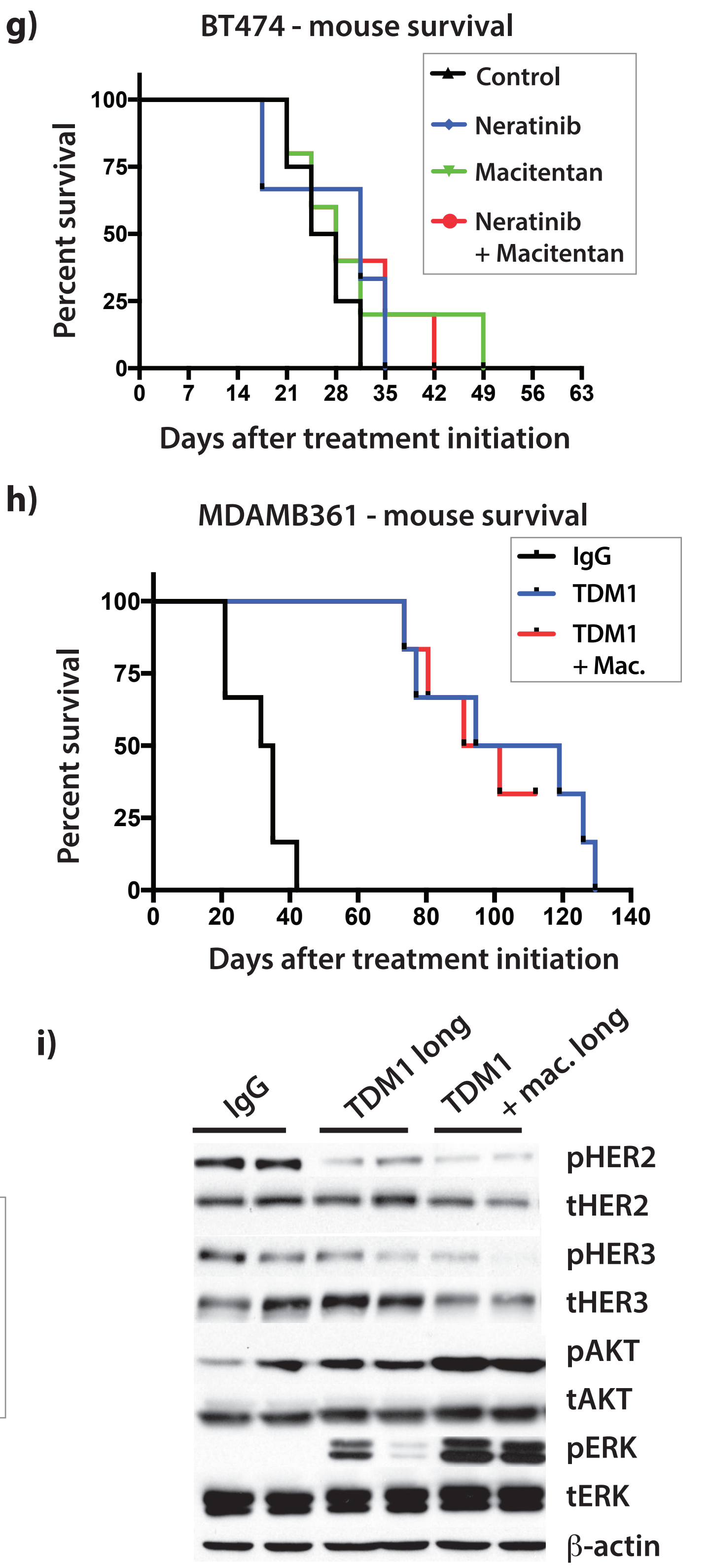

Supplement: Supplementary file 1 — Supplemental file [file 41523_2018_100_MOESM1_ESM.pdf]
